# A Novel Antioxidant, Hydrogen-Rich Coral Calcium Alters Gut Microbiome and Bile Acid Synthesis to Improve Methionine-and-Choline-Deficient Diet-Induced Non-Alcoholic Fatty Liver Disease

**DOI:** 10.3390/antiox13060746

**Published:** 2024-06-20

**Authors:** Hung-Tsung Wu, Chin-Shiang Tsai, Ting-Hsing Chao, Horng-Yih Ou, Liang-Miin Tsai

**Affiliations:** 1 Department of Internal Medicine, School of Medicine, College of Medicine, National Cheng Kung University, Tainan 701, Taiwan; z11008014@ncku.edu.tw (H.-T.W.); chaoth@mail.ncku.edu.tw (T.-H.C.); mikonlon@gmail.com (H.-Y.O.); 2 Tong-Yuan Diabetes Center, College of Medicine, National Cheng Kung University, Tainan 701, Taiwan; 3 Institute of Clinical Medicine, College of Medicine, National Cheng Kung University, Tainan 701, Taiwan; jasonmammal@gmail.com; 4 Department of Internal Medicine, National Cheng Kung University Hospital, College of Medicine, National Cheng Kung University, Tainan 704, Taiwan; 5 Department of Internal Medicine, Tainan Municipal Hospital (Managed by Show-Chwan Medical Care Corporation), Tainan 701, Taiwan

**Keywords:** antioxidant, hepatic steatosis, hydrogen-rich coral calcium, methionine and choline deficient diet, microbiome

## Abstract

The prevalence of non-alcoholic fatty liver disease (NAFLD) has dramatically increased in recent years, and it is highly associated with metabolic diseases, as well as the development of hepatocellular carcinoma. However, effective therapeutic strategies for the treatment of NAFLD are still scarce. Although hydrogen-rich water shows beneficial effects for hepatic steatosis, the inconvenience limits the application of this antioxidant. In light of this, hydrogen-rich coral calcium (HRCC) was developed due to its convenience and quantifiable characteristics. However, the effects of HRCC on NAFLD are still unknown. In the present study, we found that HRCC treatment improved methionine-and-choline-deficient diet (MCD)-induced hepatic steatosis, increased aspartate aminotransferase and alanine aminotransferase levels, and elevated hepatic inflammatory factor expressions in mice. In addition to the increased expressions of antioxidative enzymes, we found that HRCC increased the expressions of bile acid biosynthesis-related genes, including *Cyp8b1* and *Cyp27a1*. Increased hepatic bile acid contents, such as muricholic acids, 23 nor-deoxycholic acid, glycoursodeoxycholic acid, and cholic acids, were also confirmed in MCD mice treated with HRCC. Since the biogenesis of bile acids is associated with the constitution of gut microbiome, the alterations in gut microbiome by HRCC were evaluated. We found that HRCC significantly changed the constitution of gut microbiome in MCD mice and increased the contents of *Anaerobacterium, Acutalibacter, Anaerosacchariphilus,* and *Corynebacterium.* Taken together, HRCC improved MCD-induced NAFLD through anti-inflammatory mechanisms and by increasing antioxidative activities. Additionally, HRCC might alter gut microbiome to change hepatic bile acid contents, exerting beneficial effects for the treatment of NAFLD.

## 1. Introduction

In recent years, the prevalence of non-alcoholic fatty liver disease (NAFLD) has dramatically increased due to the growing obese population, and NAFLD is highly associated with metabolic diseases, including diabetes [1]. A recent meta-analysis examining NAFLD in Asia reported an incidence rate of 50.9 per 1000 person-years. Mainland China had the highest rate at 63 per 1000 person-years, while Japan had the lowest at 29 per 1000 person-years [2]. In South Korea, the NAFLD incidence was approximately 45 cases per 1000 person-years [3].

NAFLD is a spectrum ranging from simple steatosis, hepatitis, fibrosis, and cirrhosis, to the development of hepatocellular carcinoma [4]. Within this spectrum, hepatic steatosis, or fatty liver, is termed NAFL, while non-alcoholic steatohepatitis (NASH) is a more severe condition characterized by inflammation and hepatocyte damage, often accompanied by pericellular fibrosis that can progress to cirrhosis. Although imaging and clinical features, such as metabolic comorbidities and abnormal lab tests, can strongly suggest NAFL or NASH, a definitive diagnosis of NASH requires a liver biopsy. Recently, additional subgroups of NASH have been identified [5]. NASH can lead to severe liver-related outcomes like cirrhosis, liver failure, and hepatocellular carcinoma, while non-liver-related adverse outcomes are mainly linked to increased cardiovascular disease and cancer [6]. Multiple molecular pathways contribute to the development of NASH, and it is uncertain whether NASH always follows NAFL. The pathogenic mechanisms likely vary among patients, resulting in diverse clinical manifestations [7]. Although NAFLD may be multifactorial, oxidative stress is widely considered as the initiation of hepatocellular injury, leading to steatohepatitis, fibrosis, cirrhosis, and hepatocellular carcinoma [8]. Risk factors for NAFLD include poor nutrition, insufficient exercise, and inadequate sleep, with obesity being a significant risk factor. However, NAFLD has also been observed in lean individuals [9]. Another potential risk factor is the evolution of the human microbiome, influenced by dietary changes and the widespread use of antibiotics in agriculture and medicine. Evidence from mouse studies shows that a NASH phenotype can be transmitted through the microbiome [10]. Studies have found that the gut microbiome in NASH patients is less diverse than in healthy individuals, and weight loss can alter the microbiome [11,12].

Although the impact of NAFLD on human health is well-defined, effective therapeutic strategies for its treatment are still scarce [13]. In addition to weight loss and exercise, diet control is an important strategy for improving NAFLD [14]. Adequate diet intake improves NAFLD not only through calorie restriction but also through alterations in the gut microbiome [15]. It is known that metabolites from the gut microbiome play crucial roles in regulating metabolism in humans. Additionally, the gut microbiome mediates bile acid biosynthesis in the liver, and these bile acids act on the Takeda G-protein receptor 5 (TGR5) to improve NAFLD [16]. In addition, several antioxidants have reached clinical trials against NAFLD. Silymarin effectively treated NASH in mice fed a high-fat diet for 12 weeks. The treatment was effective without changes to their dietary habits [17]. Silybin, an active ingredient of silymarin, significantly reduced serum and liver fat in mice with high-fat-diet-induced NAFLD over one month and reversed metabolic disorders. It acts as a peroxisome proliferator-activated receptor (PPARα) partial agonist, contributing to its lipid-lowering effects. Silybin also ameliorated methionine-and-choline-deficient diet (MCD)-induced NAFLD in mice through PPARα activation [18,19]. Long-term vitamin C may improve adiponectin levels and reduce hepatic triglycerides and NASH risk in NAFLD patients, though the overall benefits of antioxidant vitamins in NAFLD is unclear [8].

Molecular hydrogen has been identified as an effective antioxidant therapy due to its ability to quickly diffuse across membranes. This enables it to act as an antioxidant for both preventive and therapeutic purposes by selectively reducing cytotoxic oxygen radicals without impacting other reactive oxygen species (ROS) [20]. Previous research has shown that hydrogen serves as an antioxidant, as well as an anti-apoptotic and anti-inflammatory agent, in various animal models and human clinical trials [21]. There are multiple ways to administer hydrogen, such as inhaling hydrogen gas, drinking water infused with hydrogen, and injecting saline containing hydrogen [22]. Regarding NAFLD, mice fed an MCD diet and treated with hydrogen-rich water or pioglitazone showed reduced markers of liver damage, inflammation, oxidative stress, and apoptosis. Additionally, hydrogen-rich water reduced the number and size of hepatic tumors, suggesting it may be an effective treatment for NASH by mitigating oxidative stress, inflammation, and hepatocarcinogenesis [23]. Consumption of hydrogen-rich water not only improves high-fat-diet-induced type 2 diabetes, but also NAFLD in mice due to its antioxidative and anti-inflammatory activities [24,25]. 

Although the beneficial effects of hydrogen-rich water on human health have been demonstrated for the past few years [26], and the effects of hydrogen water on the improvement of high-fat-diet-induced metabolic diseases have been well-defined, the inconvenience and unstable characteristics limit its application. Regarding this issue, hydrogen-rich coral calcium (HRCC) was developed. Coral calcium, derived from coral exoskeletons, mainly consists of calcium carbonate (CaCO_3_, 20%) and magnesium (Mg, 10%). An earlier study found that Mg-doped CaCO_3_ powder released significantly more hydrogen after high-pressure hydrogen loading compared with calcitic CaCO_3_ [27]. By immobilizing hydrogen on the surface of coral calcium, HRCC can be created, providing a safer and more convenient method of hydrogen delivery [28]. Although it is known that HRCC exerts a stable antioxidative capacity and shows improvement of alcoholic intoxication and impaired liver function [29], the effects of HRCC on NAFLD are still unknown. 

In the present study, an MCD-induced NAFLD mice model was established to evaluate the efficacy of HRCC for improving NAFLD. Not only were the effects of HRCC on antioxidative enzyme expressions determined, but also the effects of HRCC on the alteration of the gut microbiome and hepatic bile acid contents were clarified

## 2. Materials and Methods

### 2.1. Hydrogen-Rich Coral Calcium

Porous coral materials and HRCC were purchased from HoHo Biotech Co., Ltd. (Taipei, Taiwan), and the method for the production of HRCC was illustrated in previous studies [28,30]. Briefly, the porous coral materials were sealed in a high-pressure and high-temperature chamber filled with 100% hydrogen gas. After thermal expansion, HRCC was generated and sterilized. The powder was further obtained by grinding. 

### 2.2. Animals

**Figure 1:**
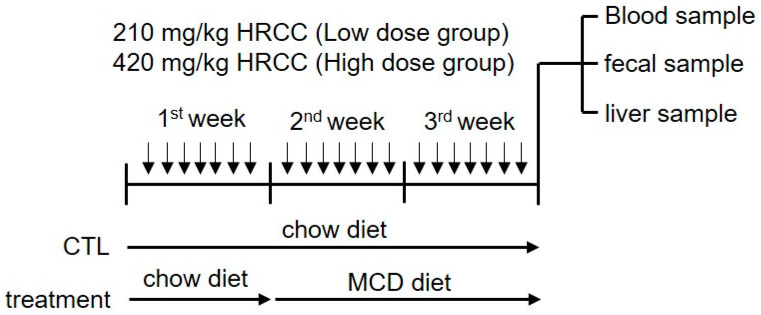
Flowchart of the experimental procedure. Non-alcoholic fatty liver disease was induced in C57BL/6 mice by feeding with a methionine-and-choline-deficient diet (MCD) for two weeks, and the animals were randomly assigned into four groups (N = 5–8 for each group). Mice were pre-treated once daily with indicated doses of hydrogen-rich coral calcium (HRCC) by oral gavage for one-week, and then NAFLD was induced by feeding the mice with MCD for another two weeks. The body weight of each group of mice was recorded every week. At the end of the experiment, the fecal samples were collected using metabolic cages, and the blood and liver samples were harvested for further experiments.

Eight-week-old C57BL/6 male mice were obtained from the Laboratory Animal Center, College of Medicine at National Cheng Kung University, and housed in an environment at a temperature of 23 ± 2 °C, humidity 60 ± 10%, and alternating light and dark cycles every 12 h (lights on at 07:00AM). The mice were randomly assigned to four groups (N = 5–8 for each group), including [1] paired-control diet (A02082003BY, Research Diets, New Brunswick, NJ, USA)-fed mice treated with 210 mg/kg porous coral calcium dissolved in 3% carboxymethyl cellulose (Sigma-Aldrich, St. Louis, MO, USA) (CC group) via oral gavage; [2] MCD-fed (A02082002BR, Research Diets) mice treated with 210 mg/kg porous coral calcium (MC group); [3] MCD-fed mice treated with 210 mg/kg HRCC (low-dose group; ML); and [4] MCD-fed mice treated with 420 mg/kg HRCC (high-dose group; MH). After the pre-treatment of coral calcium or previously indicated doses of HRCC once daily for one week, NAFLD was induced by feeding the mice with MCD for another two weeks, and the body weight of each group of mice was recorded every week. At the end of the experiment, each group of mice was anaesthetized and then the blood and liver samples were harvested for further experiments (Figure 1). Serum aspartate aminotransferase (AST) and alanine aminotransferase (ALT) (Abcam, Cambridge, UK), as well as serum endotoxin levels (Thermo Scientific, Lenexa, KS, USA), were determined using commercialized assay kits.

### 2.3. Hematoxylin and Eosin Stain 

At the end of the experiments, the liver tissues from each group of mice were removed and then fixed in 10% formalin. Five-μm thick sections were deparaffinized, and stained with hematoxylin for 3–8 min and eosin solution for another 1–3 min. After dehydration, the sections were sealed and observed under a light-field microscope.

### 2.4. Western Blot Analysis

The protein samples from liver tissues were extracted and mixed with radioimmunoprecipitation lysis buffer (VWR Chemical, Solon, OH, USA), containing protease inhibitors (Sigma-Aldrich, St. Louis, MO, USA). After centrifugation at 13,000 rpm at 4 °C for 10 min, the supernatant was collected and the protein concentration was determined using a bicinchoninic acid assay kit (Visual Protein, Taipei, Taiwan). The proteins were separated by 10% sodium dodecyl sulfate-polyacrylamide gel electrophoresis, and then transferred to polyvinylidene difluoride membranes (Biomate, Taipei, Taiwan). The membranes were blocked with 10% skim milk for one hour at room temperature, and then incubated with a 1:1000 dilution of primary antibodies (Table 1), including superoxide dismutase [Cu-Zn] (SOD1; Novus Biologicals, Centennial, CO, USA), glutathione peroxidase-1 (GPx; Abcam), catalase (Cell Signaling Technology, Danvers, MA, USA), or TGR5 (Abcam) at 4 °C overnight. α-Tubulin was used as an internal control (Abcam). Afterwards, the membranes were washed with Tris-buffered saline with Tween 20 (TBS-T) (10 mM Tris (pH 7.6), 150 mM NaCl, and 0.05% Tween 20), and then the blots were incubated with a 1:5000 dilution of horseradish peroxidase-conjugated secondary antibodies at room temperature for one hour. The protein bands were detected using Immobilon (Millipore, Billerica, MA, USA), and the signal intensity was quantified using ImageJ software (https://imagej.nih.gov/nih-image/, accessed on 1 October 2021).

### 2.5. Ribonucleic Acid (RNA) Extraction

RNA from each sample was extracted using GENEzol reagent (Geneaid Biotech, New Taipei City, Taiwan), according to the manufacturer’s instruction. Briefly, 100 mg of liver sample was homogenized with 1 mL GENEzol Reagent and 200 μL chloroform. After vigorously vortex for 10 s, the samples were centrifuged at 12,000× *g* for 15 min at 4 °C to separate the phases. The upper aqueous phase was mixed with 1 mL isopropanol for 10 min at room temperature, and then centrifuged at 12,000× *g* for 10 min at 4 °C. After careful removal of the supernatant, the RNA pellet was washed with 70% ethanol, air-dried for 5–10 min at room temperature, and then resuspended with RNase-free water.

### 2.6. Real-Time Quantitative Polymerase Chain Reaction

The gene expressions of proinflammatory cytokines (Table 2), including interleukin-6 (Il6), tumor necrosis factor alpha (Tnfa), the C-C motif chemokine ligand 2 (Ccl2), cytochrome P450 7a1 (Cyp7a1), cytochrome P450 8b1 (Cyp8b1), and cytochrome P450 27a1 (Cyp27a1) in mouse liver, were examined using real-time quantitative polymerase chain reaction. Reverse transcription was performed to generate complementary DNA using the MMLV Reverse Transcription Kit (Protech, Placentia, CA, USA), and the real-time fluorescence quantitative polymerase chain reaction instrument (StepOnePlus Real-Time PCR Detection System, Applied Biosystems, Foster City, CA, USA) was used to quantify relative messenger RNA expression levels with the 2^−ΔΔct^ method. 

### 2.7. Gut Microbiome Analysis by Full-Length 16S Ribosomal RNA Sequencing

Full-length 16S ribosomal RNA sequencing was used to analyze the gut microbiome (Toolsbiotech, Xizhi, New Taipei City, Taiwan). Briefly, 250 mg fecal matter from each group of mice was collected in metabolic cages during an overnight fast. Total genomic deoxyribonucleic acid from the fecal samples was extracted using the column-based method (QIAamp PowerFecal DNA Kit, Qiagen, Hilden, Germany). The full-length 16S genes (V1–V9 regions) were amplified by barcoded 16S gene-specific primers, and the SMRTbell library was prepared according to the amplification of the full-length 16S gene. The feature-classifier and classify-consensus-vsearch algorithm in QIIME2 (v2022.11) were employed to annotate taxonomy classification based on the information retrieved from the National Center for Biotechnology Information 16S ribosomal RNA database. 

### 2.8. Determination of Bile Acid Content in the Liver

Twenty milligrams of each sample were extracted with 1 mL extraction buffer (methanol:acetonitrile:H_2_O:formic acid = 2:2:1:0.8) containing an internal standard mixture. After vortex for 30 s, the samples were homogenized at 35 Hz for 4 min and sonicated for 5 min in an ice-cold water bath. Then, the samples were incubated for one hour at −20 °C and centrifuged at 12,000 rpm for 15 min at 4 °C. The supernatant was then transferred for bile acid analysis. The analysis was performed using Waters ultra-high performance liquid chromatography coupled with Waters Xevo TQS MS (Waters Corp, Milford, MA, USA). Briefly, the chromatographic separation was performed on a Waters ACQUITY BEH C8 column (2.1 mm × 100 mm × 1.7 mm) at 60 °C. The mobile phase A was 10% acetonitrile with 0.01% formic acid and the mobile phase B was isopropanol/acetonitrile (50:50, *v*/*v*) with 0.01% formic acid. Mass analysis was performed using the Waters Xevo TQ-S system in positive-ion electrospray ionization mode, and the capillary voltage was set at 1.5 KV. The desolvation gas flow rate was set at 1000 L/h, and cone gas flow was maintained at 150 L/h. The desolvation and source temperatures were set at 600 °C and 150 °C, respectively. A QC sample (laboratory quality control) and mix QC sample (a mixture of all samples) were prepared and analyzed during the analytical runs after every 10th sample. The data were then processed using TargetLynx to integrate signal strength and convert it to concentration.

### 2.9. Statistics

Graphpad prism 8 was used for illustration and statistical analyses. The data were presented as mean ± standard error (SEM). Student’s *t*-test or one-way ANOVA followed by Tukey’s post hoc test were used. For statistical analysis, significance of all species among groups at various taxonomic levels was detected using differential abundance analysis with a zero-inflated Gaussian (ZIG) log-normal model, as implemented in the “fitFeatureModel” function of the R Bioconductor metagenomeSeq package. Beta-diversity among groups was analyzed using PERMANOVA and constrained principal coordinates analysis (cPCoA). The Wilcoxon rank-sum test was used to compare relative abundance of bacteria differences among groups at the family level. Statistically significance was defined as *p* < 0.05. 

## 3. Results

### 3.1. Administration of HRCC Improved MCD-Induced Weight Loss in Mice

**Figure 2:**
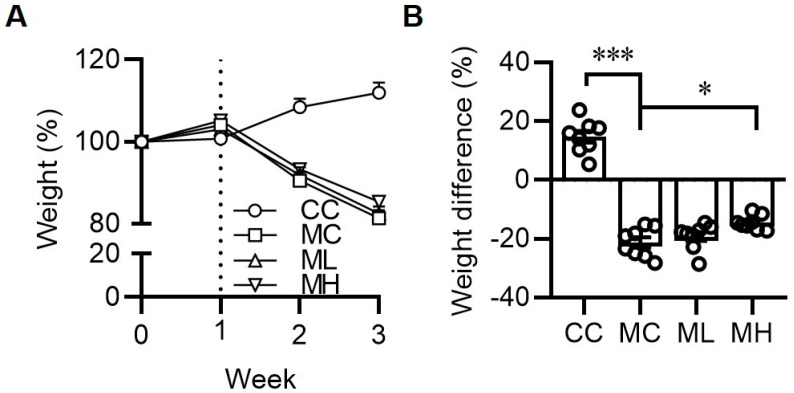
Consumption of hydrogen-rich coral calcium (HRCC) improved methionine-and-choline-deficient diet-induced weight loss. C57BL/6 mice were pre-treated by oral gavage with 210 mg/kg HRCC (low-dose group, ML), 420 mg/kg HRCC (high-dose group; MH), or coral calcium (control group; CC) for seven days. After the pre-treatment, the mice were fed with a methionine-and-choline-deficient diet (MC) for another two weeks to induce non-alcoholic fatty liver disease, the body weight of each group of the mice was recorded every week (**A**), and the changes in body weight were calculated (**B**). N = 6–8 mice in each group; * *p* < 0.05; *** *p* < 0.001 as compared with indicated groups.

In mice fed with an MCD diet, the body weight significantly decreased after one week of feeding. However, the group of mice fed with a high dose of HRCC showed a slight and significant improvement in MCD-induced body-weight loss (Figure 2A,B).

**Figure 3:**
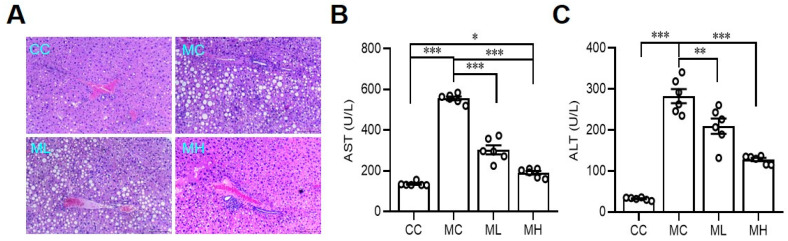
Hydrogen-rich coral calcium (HRCC) consumption improved methionine-and choline-deficient-diet-induced non-alcoholic fatty liver disease. C57BL/6 mice were pre-treated by oral gavage with 210 mg/kg HRCC (low-dose group, ML), 420 mg/kg HRCC (high-dose group; MH), or coral calcium (control group; CC) for seven days. After the pre-treatment, the mice were fed with a methionine-and-choline-deficient diet (MC) for another two weeks to induce non-alcoholic fatty liver disease. At the end of the experiments, the animals were sacrificed and the liver sections were collected for the determination of fatty liver (magnification 200×) (**A**). The blood samples were collected for the measurement of aspartate aminotransferase (AST) (**B**) and alanine aminotransferase (ALT) (**C**) concentrations. N = 6–8 mice in each group; * *p* < 0.05; ** *p* < 0.01; *** *p* < 0.001 as compared with indicated groups.

Additionally, MCD-induced lipid accumulation in the liver significantly decreased in the group of mice fed with a high dose of HRCC compared with the MCD group (Figure 3A). Moreover, MCD-induced liver dysfunction, as determined by elevated serum levels of AST (Figure 3B) and ALT (Figure 3C), was improved in both low-dose and high-dose HRCC-treated groups.

### 3.2. Consumption of HRCC Decreased MCD-Induced Hepatic Inflammation in Mice

**Figure 4:**
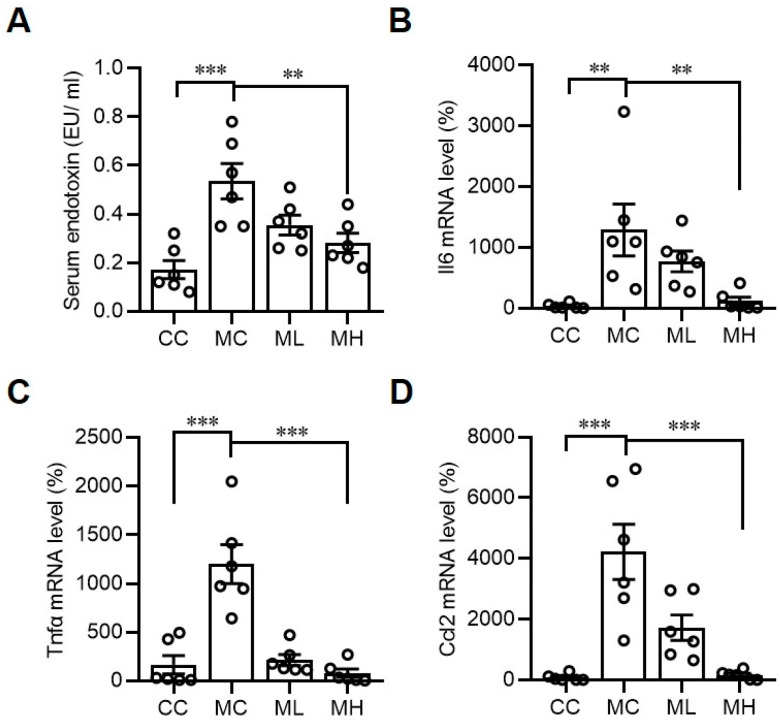
Hydrogen-rich coral calcium (HRCC) consumption improved methionine-and-choline-deficient diet-induced inflammation. C57BL/6 mice were pre-treated by oral gavage with 210 mg/kg HRCC (low-dose group, ML), 420 mg/kg HRCC (high-dose group; MH), or coral calcium (control group; CC) for seven days. After the pre-treatment, the mice were fed with a methionine-and-choline-deficient diet (MC) for another two weeks to induce non-alcoholic fatty liver disease. At the end of the experiments, the blood samples were collected for the determination of serum endotoxin concentrations (**A**), and the liver tissues were removed for the determination of interleukin-6 (Il6) (**B**), tumor necrosis factor-α (Tnfa) (**C**), and C-C motif chemokine ligand 2 (Ccl2) (**D**) gene expression levels by quantitative-polymerase chain reaction. N = 6 for each group of mice; ** *p* < 0.01; *** *p* < 0.001 as compared with indicated groups.

In view of the anti-inflammatory activities of hydrogen molecules, we then investigated the effects of HRCC on the expressions of proinflammatory factors. Consistent with a previous study that showed that MCD increased serum endotoxin levels in mice [31], administration of HRCC in MCD mice significantly decreased serum endotoxin levels (Figure 4A). We also found that the gene expressions of proinflammatory factors in the liver, including Il6 (Figure 4B), Tnfa (Figure 4C), and Ccl2 (Figure 4D), were significantly decreased, indicating HRCC has an anti-inflammatory activity. 

### 3.3. Consumption of HRCC Increases Hepatic Antioxidative Enzyme Expressions in MCD-Induced NAFLD Mice

**Figure 5:**
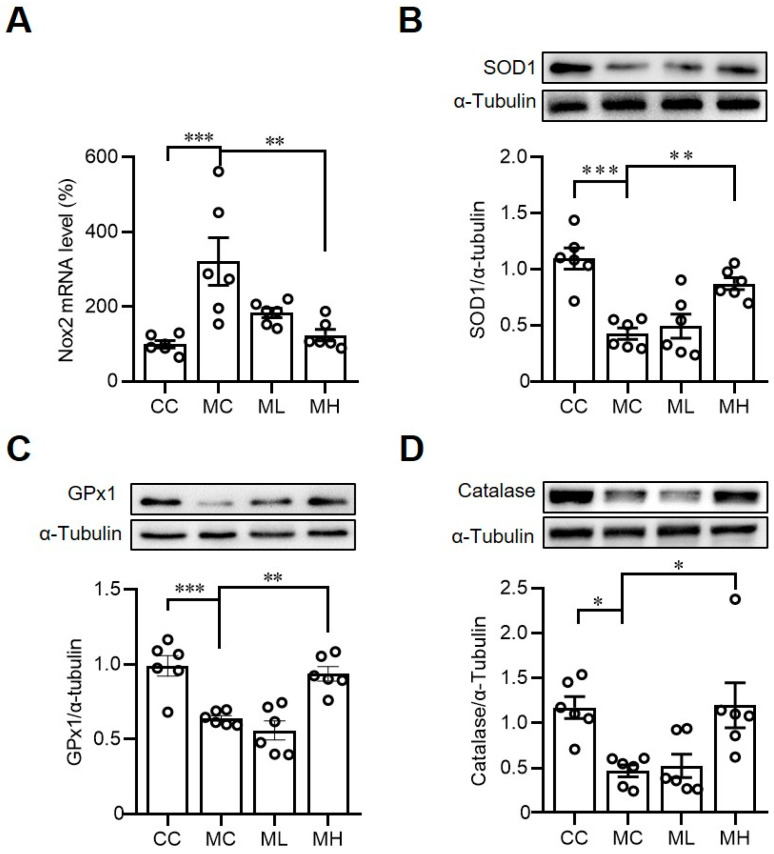
Consumption of hydrogen-rich coral calcium (HRCC) increased the hepatic expressions of oxidative stress-related enzymes. C57BL/6 mice were pre-treated by oral gavage with 210 mg/kg HRCC (low-dose group, ML), 420 mg/kg HRCC (high-dose group; MH), or coral calcium (control group; CC) for seven days. After the pre-treatment, the mice were fed with a methionine-and-choline-deficient diet (MC) for another two weeks to induce non-alcoholic fatty liver disease. At the end of the experiments, the liver tissues were removed for the determination of NADP oxidase 2 (Nox2) gene expression by quantitative-polymerase chain reaction (**A**). In addition, manganese superoxide dismutase (SOD1) (**B**), glutathione peroxidase-1 (GPx) (**C**), and catalase (**D**) protein expressions were evaluated by Western blots. N = 6 mice for each group; * *p* < 0.05; ** *p* < 0.01; *** *p* < 0.001 as compared with indicated groups.

Due to the antioxidative activities of the hydrogen molecule, the effects of HRCC on the hepatic expressions of antioxidative enzymes were also evaluated. As shown in Figure 5, we first examined the oxidative stress in MCD mice by *Nox2*, which encoded a superoxide-generating enzyme to form ROS. Consistent with a previous study [31], an MCD diet significantly increased the gene expression of the oxidative marker, *Nox2*; however, treatment with HRCC reversed MCD-induced Nox2 expressions, indicating HRCC had an effect in decreasing oxidative stress (Figure 5A). We then evaluated the protein expressions of antioxidative enzymes, and we found that MCD consumption significantly decreased the expressions of antioxidative enzymes. However, treatment with HRCC increased the expressions of hepatic antioxidative enzymes, including SOD1 (Figure 5B), GPx (Figure 5C), and catalase (Figure 5D). 

### 3.4. Consumption of HRCC Increases Bile Acid Biogenesis Gene Expressions and Hepatic Bile Acid Content in MCD-Induced NAFLD Mice

**Figure 6:**
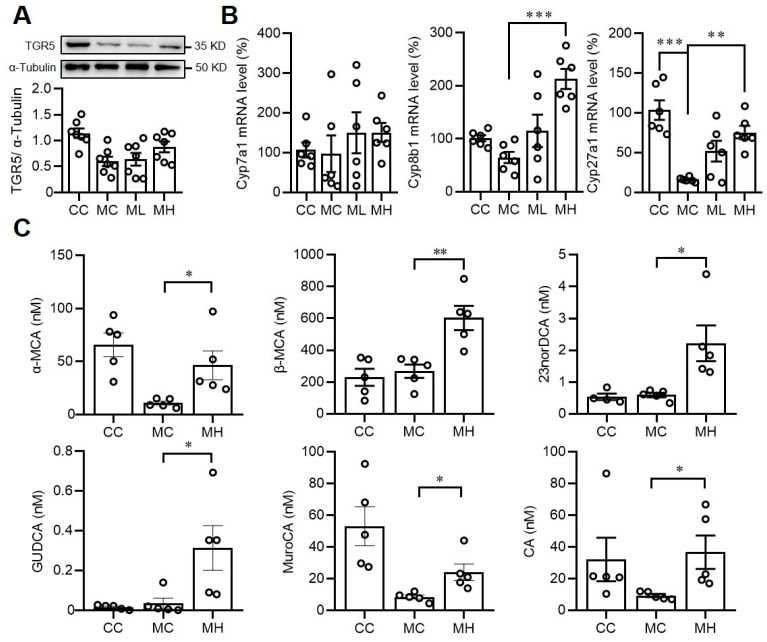
Consumption of hydrogen-rich coral calcium (HRCC) altered the hepatic expressions of bile acid biogenesis-related genes and bile acid contents. C57BL/6 mice were pre-treated by oral gavage with 210 mg/kg HRCC (low-dose group, ML), 420 mg/kg HRCC (high-dose group; MH), or coral calcium (control group; CC) for seven days. After the pre-treatment, the mice were fed with a methionine-and-choline-deficient diet (MC) for another two weeks to induce non-alcoholic fatty liver disease. At the end of the experiments, the liver tissues were removed for the determination of Takeda G-protein receptor 5 (TGR5) protein levels by Western blots (**A**). Moreover, the bile acid biogenesis-related gene expressions in the liver of the mice were determined by real time-PCR (**B**). Furthermore, the bile acids were extracted from the liver of each group of the mice, and then quantified using gas chromatography-mass spectrometry (**C**). N = 5–7 mice for each group; * *p* < 0.05; ** *p* < 0.01; *** *p* < 0.001 as compared with indicated groups.

It is known that activation of TGR5 by bile acids improves NAFLD, so we therefore investigated the effects of HRCC on TGR5 expressions. We found that HRCC consumption showed no significant effects on hepatic TGR5 expression (Figure 6A). However, treatment with HRCC at high doses (MH group) either increased the expression of bile acid biosynthesis-related genes such as *Cyp8b1* in MCD-fed mice or reversed the MCD diet-induced suppression of the expressions of bile acid biosynthesis-related genes such as *Cyp27a1* (Figure 6B), implying HRCC might alter bile acid content in MCD mice. We then investigated the hepatic bile acid content in MCD mice and confirmed that the hepatic bile acid contents, such as α- and β-muricholic acids (MCA), 23 nor-deoxycholic acid (23 norDCA), glycoursodeoxycholic acid (GUDCA), murocholic acid (MuroCA), and cholic acid (CA), were increased in MCD mice treated with HRCC (Figure 6C). 

### 3.5. Consumption of HRCC Alters Gut Microbiome Composition in MCD-Induced NAFLD Mice

**Figure 7:**
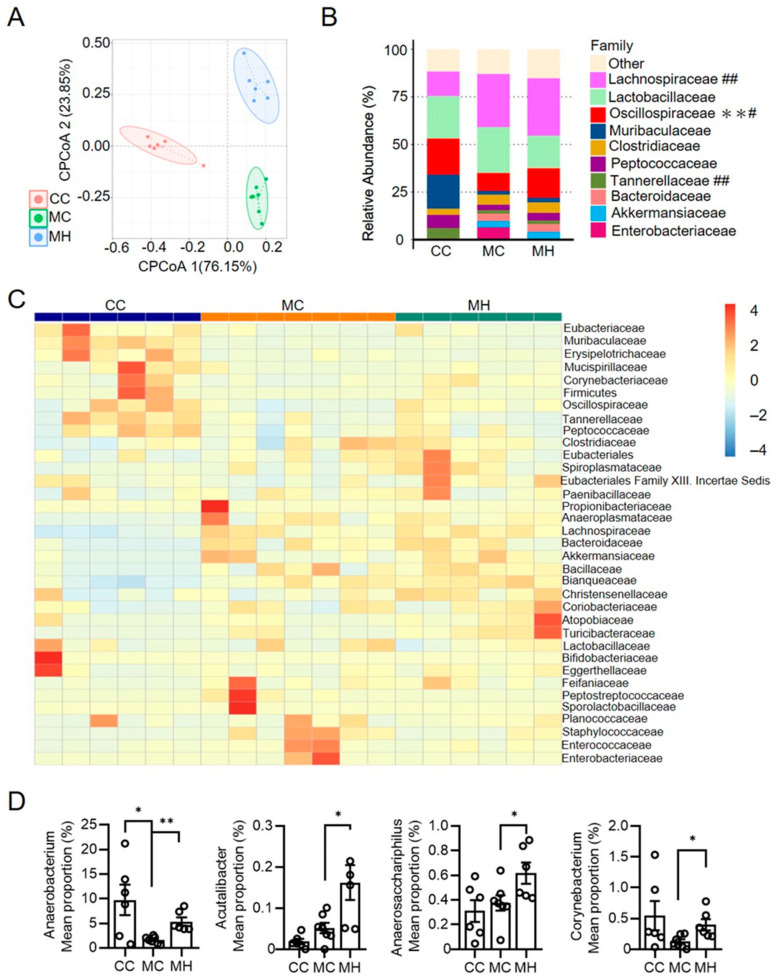
Consumption of hydrogen-rich coral calcium (HRCC) altered the composition of gut microbiome. C57BL/6 mice were pre-treated by oral gavage with 420 mg/kg HRCC (high-dose group; MH), or coral calcium (control group; CC) for seven days. After the pre-treatment, the mice were fed with a methionine-and-choline-deficient diet (MC) for another two weeks to induce non-alcoholic fatty liver disease. The feces of each group of mice were collected, and gut microbiome composition by principal co-ordinates analysis (**A**), relative abundance in the family (**B**,**C**), and genus (**D**) in the gut microbiome using 16S sequencing were determined. N = 6 mice for each group; * *p* < 0.05; ** *p* < 0.01 as compared with CC group or indicated groups. ^#^ *p* < 0.05; ^##^ *p* < 0.01 as compared with MC group.

Since the biogenesis of bile acids is associated with the constitution of the gut microbiome, the alterations of the gut microbiome by HRCC was evaluated. We found that treatment with HRCC significantly changed the constitution of the gut microbiome in MCD mice (Figure 7A). Among the changed families in the gut microbiome, *Lachnospiraceae*, *Oscillospiraceae*, and *Tannerellaceae* showed significant alterations in abundance after treatment with HRCC in MCD mice (Figure 7B,C). We further investigated the changes in the genus in the gut microbiome, and we found that the proportion of *Anaerobacterium*, *Acutalibacter*, *Anaerosacchariphilus*, and *Corynebacterium* were significantly increased after treatment with HRCC in MCD mice (Figure 7D). 

## 4. Discussion

To the best of our knowledge, the present study is the first to investigate the effects of HRCC on the improvement of NAFLD. We successfully established a mouse model of NAFLD induced by an MCD diet to assess the effectiveness of HRCC in improving NAFLD. We not only investigated the effects of HRCC on the expression of inflammatory cytokines and antioxidative enzymes but also explored its impact on the changes in the gut microbiome and hepatic bile acid composition. Our previous study unveiled that HRCC could not only improve alcohol intoxication, but could also significantly reduce hepatic inflammation. Since there is already evidence indicating a strong correlation between the above inflammation markers and NAFLD [32,33,34], it is plausible that HRCC may not only be beneficial in improving alcohol intoxication, but should also be of assistance in ameliorating NAFLD. 

Oxidative stress arises from an imbalance between oxidants and antioxidants. ROS are notably harmful and necessitate detoxification through an antioxidant system comprised of various enzymes that eliminate ROS [35]. Among the pertinent enzymes are SOD, catalase, and GPx. The literature has demonstrated an elevation in oxidative stress levels among NAFLD patients compared with healthy individuals, with the potential for even greater severity than in patients with chronic viral hepatitis [36,37]. The present study using an animal model reveals that HRCC has the capacity to alleviate the oxidative stress associated with NAFLD, suggesting its potential as an antioxidant-based therapy for NAFLD treatment. In addition, since the coral calcium without hydrogen was used as a control group, the improvement of NAFLD by HRCC can be supposed due the enrichment of hydrogen. Also, increased hydrogen levels can be detected in exhaled gas from rats gavaged with HRCC [30], and the antioxidative activity of HRCC might be a systemic effect in mice.

Bile acids play a pivotal role in regulating the overall balance of cholesterol in the body, and act as signaling molecules and metabolic regulators, through activating TGR5 [38]. Since we found that the expression of TGR5 showed no significant changes after the treatment with HRCC in MCD mice, we further examined the levels of hepatic bile acids. The interplay between the gut and liver is critically important in converting primary bile acids into secondary bile acids, maintaining bile acid composition in the pool, and regulating metabolic balance to prevent conditions such as hyperglycemia, dyslipidemia, obesity, and diabetes [39]. In the present study, mice subjected to an MCD diet exhibited alterations in hepatic gene expression associated with bile acid synthesis, leading to a suppression of bile acid synthesis. Treatment with HRCC might alter bile acid content in MCD mice by either increasing Cyp8b1 expression or reversing the MCD diet-induced suppression of Cyp27a1 expression.

Cyp27a1, the gene encoding the key enzyme in the alternative pathway of bile acid synthesis, has the activity to convert CDCA into α-MCA, and UDCA into β-MCA [40]. It is known that an increase in Cyp7a1 expression effectively reduces hepatic free cholesterol and oxidative stress, and reverses hepatic inflammation and fibrosis in MCD diet-fed Cyp7a1 knockout mice [41]. Although we found that the expression of Cyp7a1 showed no significant changes after the treatment with HRCC in MCD mice, the expressions of Cyp8b1 and Cyp27a1were elevated. Overexpression of Cyp27a1 in Kupffer cells reduces hepatic inflammation [42]. Of note, our results also showed increased levels of GUDCA and 23-norDCA levels in MCD mice treated with HRCC. A previous study showed that GUDCA improves diabetes and other metabolic conditions, linked with the regulation of the bile acid and gut microbiota composition [43]. Another study revealed reduced 23-norDCA is association with pediatric NAFLD [44]. The present study aligns with prior research indicating that bile acid synthesis may lead to the mitigation of hepatic inflammation, whereas diminished bile acid synthesis exacerbates MCD-induced hepatic inflammation and fibrosis [45]. These findings have contributed to the understanding of the regulatory mechanisms underlying hepatic responses to MCD-induced fatty liver, offering potential therapeutic options targeting bile acid synthesis pathways. A significant role of the gut microbiota is converting primary bile acids into secondary bile acids. 

Previous investigations have established an association between alterations in gut microbiota and some infectious diseases, immune dysregulation, and obesity [46], and gut microbiota have a significant impact on bile acids [47,48]. The gut microbiota not only oversees the metabolism of secondary bile acids but also reduces liver lipid synthesis by easing Farnesoid X receptor inhibition [49]. Research in both mice and humans indicates that bile acid transformation by the gut microbiota (including deconjugation, dehydrogenation, and dehydroxylation) is linked to the progression of NAFLD and NASH [50]. Another study found that pediatric NAFLD is linked to two key changes in the intestinal microbiome: a decline in α-diversity and increased person-to-person variability in microbiome composition. The reduction in α-diversity, indicating fewer different microbial taxa, did not correspond to a decrease in metabolic potential, unlike findings in adult obesity studies. Increased person-to-person variability was strongly associated with differences in microbial metabolic potential, aligning with trends observed in other microbiome-related disorders under various stressors [51].

Patients with NAFLD exhibit distinct microbial signatures, including increased *Proteobacteria* and *Enterobacteriaceae*, and decreased *Rikenellaceae* and *Ruminococcaceae*, as compared with healthy controls. At the genus level, there is an increase in *Escherichia*, *Dorea*, and *Peptoniphilus*, and a decrease in *Anaerosporobacter*, *Coprococcus*, *Eubacterium*, *Faecalibacterium*, and *Prevotella*. Despite these findings, studies show large discrepancies in microbial changes at various taxonomic levels. Research specifically on hepatic fibrosis is limited, but consistent patterns emerge [52]. Patients with less severe liver alterations or healthy controls have decreased Gram-negative bacteria and *Fusobacteria*, and increased Gram-positive bacteria, *Firmicutes*, and *Prevotella,* as compared with those with advanced fibrosis [53]. In the present study, we found that HRCC effectively changed NAFLD-associated gut microbiota signatures. HRCC increased the content of *Oscillospiraceae* and *Peptococcaceae* at the family level, and *Anaerobacterium* (belonging to the family *Oscillospiraceae),* as well as non-signature *Corynebacterium* (belonging to the family *Corynebacteriaceae*) at the genus level, while NAFLD signature *Enterobacteriaceae* showed an inverse trend in HRCC-treated MCD mice. However, certain NAFLD signatures in the MH group did not manifest significant changes, including *Lachnospiraceae*, *Clostridiaceae*, *Bacteroidceae*, and *Akkmanisiaceae* [54,55,56], as compared with the MC group. These results indicate distinctions in the microbiota of the MH group, as compared with both CC and MC groups. Furthermore, although prior mouse model studies and fecal transplantation experiments show that gut microbiota play a causal role in NAFLD development, as demonstrated by fecal microbiota transplantation leading to liver steatosis and inflammation [57], the results should be cautiously interpreted, since mouse models have limitations in replicating the full spectrum of human NAFLD histological lesions and associated conditions like being overweight and insulin resistance, due to differences in gut microbiota composition and digestive tract architecture between mice and humans [53].

**Figure 8:**
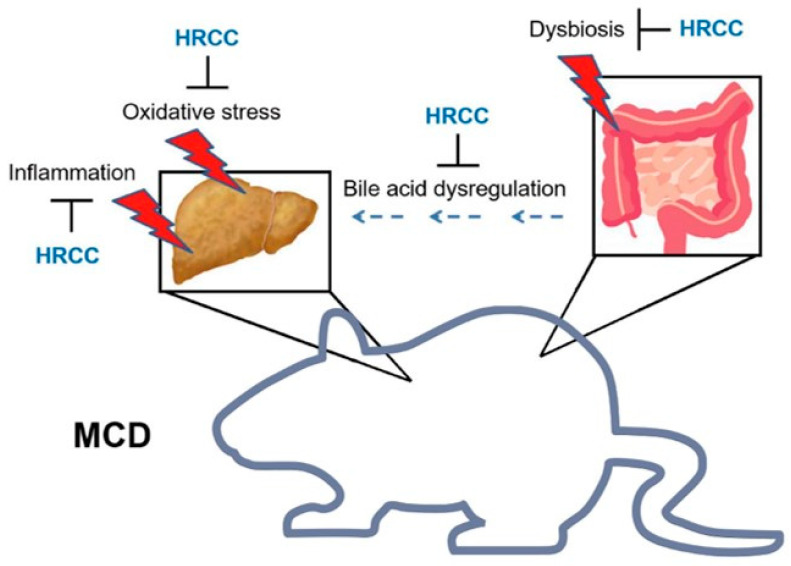
Possible mechanisms of hydrogen-rich coral calcium (HRCC) in improving methionine-and-choline-deficient diet (MCD)-induced non-alcoholic fatty liver disease (NAFLD). Administration of HRCC in mice improved MCD-induced hepatic inflammation and oxidative stress, and altered gut microbiome to change bile acid content. These beneficial effects might further improve MCD-induced NAFLD.

NAFLD is a complex disease affected by genetic, epigenetic, and environmental factors, and its exact mechanisms remain unclear. Currently, the “multiple-hit” hypothesis offers a more accurate explanation. Among all factors associated with NAFLD, oxidative stress is seen a major contributing factor [27]. A prior study of zebra fish model showed the trends of oxidative stress biomarkers were associated with gut microbiota, especially at the genus level [58]. Given the prevailing perspective on the nature of NAFLD involving the multiple-hit hypothesis [59], we infer that the impact of HRCC is comprehensive, significantly altering the gut microbiota, while not all signatures in the gut microbiota have a pivotal impact on the pathogenesis of NAFLD. We speculate that HRCC may reverse dysbiosis and affect bile acid synthesis through the expression of Cyp8b1 and Cyp27a1, treating NAFLD, together with alleviation of oxidative stress (Figure 8). This study indicates that HRCC may potentially impact bile acid and gut microbiota while simultaneously improving NAFLD, and the metabolites of the intestinal flora might be involved in HRCC-improved NAFLD in MCD mice and need further analysis to investigate. 

## 5. Conclusions

HRCC exerts anti-inflammation and antioxidation activities and might alter the gut microbiome to change bile acid content, hence improving MCD-induced NAFLD (Figure 8). The interplay between these beneficial effects still warrants further elucidation. Although HRCC might be a potential supplement for the treatment of NAFLD, the effects of HRCC in humans are still unknown. Thus, it may be worthwhile to conduct clinical trials with long-term longitudinal follow-up for more solid evidence.

## Figures and Tables

**Table 1:** Antibodies used for Western blots.

Antibody	Molecule Weight (KiloDalton, KD)	Company, Catalog Number
SOD1	16	Novus, NBP1-31204
GPx	22	Abcam, ab108427
catalase	60	Cell signaling, 14097
TGR5	35	Abcam, ab72608
α-Tubulin	50	Abcam, ab7291

Abbreviations: superoxide dismutase [Cu-Zn] (SOD1), glutathione peroxidase-1 (GPx), Takeda G protein-coupled receptor 5 (TGR5).

**Table 2:** Primers used in the present study for the determination of proinflammatory cytokines.

Gene	Forward	Reverse
Il6	AGTTGCCTTCTTGGGACTGA	TCCACGATTTCCCAGAGAAC
Tnfa	CCCTCACATCAGATCATCTTCT	GCTACGACGTGGGCTACAG
Ccl2	CCACTCACCTGCTGCTACTCA	TGGTGATCCTCTTAGCTCTCC
Nox2	ACTCCTTGGGTCAGCACTGG	GTTCCTGTCCAGTTGTCTTCG
Cyp7a1	ACAACTAAACAACTGCCATACTA	GTCCGGATATTCAAGGATGCA
Cyp8b1	ACGCTTCCTCTATCGCCTGAA	GTGCCTCAGACGCAGAGGAT
Cyp27a1	CGGGGACCGGAACGCTAC	AGTCCCAAAGGAGGTTGTCCA
18s	CATGGCCGTTCTTAGTTGGTGG	CGCTGAGCCAGTCAGTGTAG

Abbreviations: interleukin-6 (Il6), tumor necrosis factor alpha (Tnfa), the C-C motif chemokine ligand 2 (Ccl2), NADP oxidase 2 (Nox2), cytochrome P450 7a1 (Cyp7a1), cytochrome P450 8b1 (Cyp8b1), and cytochrome P450 27a1 (Cyp27a1)

## Data Availability

Dataset available on request from the authors.
